# Ultraprocessed Food and Risk of Cancer: Mechanistic Pathways and Public Health Implications

**DOI:** 10.3390/cancers17132064

**Published:** 2025-06-20

**Authors:** Bruna Menegassi, Manlio Vinciguerra

**Affiliations:** 1 Sociology and Communication Department, Social Sciences Faculty, Salamanca University, 37008 Salamanca, Spain; menegassi@usal.es; 2 Department of Translational Stem Cell Biology, Research Institute, Medical University of Varna, 9002 Varna, Bulgaria; 3 School of Pharmacy and Biomolecular Sciences, Liverpool John Moores University, Liverpool L3 3AF, UK

**Keywords:** ultraprocessed foods, cancer risk, food additives, public health, nutritional epidemiology

## Abstract

Ultraprocessed foods (UPFs), now dominant in global diets, pose health risks that go beyond poor nutrition due to the synergistic effects of compounds in their ultracomplex industrial formulations. This narrative review aims to provide researchers and health professionals in the field of cancer with updated and critical information, as they are often unaware of the complex and evolving evidence linking UPFs to carcinogenesis. The review discusses potential mechanisms through which UPFs may contribute to cancer development, including harmful additives, neo-formed contaminants, and packaging-derived substances, as well as the displacement of protective nutrients found in whole foods. Despite limitations in establishing direct causality, epidemiological studies consistently associate high UPF intake with increased incidence of various cancers, notably colorectal, breast, and pancreatic cancers. These findings reflect a broader paradigm shift in nutritional epidemiology, recognizing that food processing is an essential dimension of diet-related health risks. To mitigate the impact of UPFs, the review emphasizes the need for preventive strategies that integrate clear dietary guidelines, regulatory measures on food labeling and additives, and public education campaigns. Successful international experiences in regulating marketing and improving transparency serve as important references. Moreover, eliminating corporate influence and conflicts of interest is crucial to ensure that public health, rather than industry agendas, guides nutrition policy. As scientific research advances to clarify the mechanisms of action and synergistic effects of harmful compounds in UPFs, coordinated efforts are needed to reduce their consumption and ultimately alleviate the global cancer burden.

## Simple Summary

Ultraprocessed foods (UPFs) are common in today’s diets and have been linked to serious health problems, including cancer. This review examines current research on how UPFs may increase cancer risk and highlights the need for stronger prevention strategies. By examining how UPFs are made and what harmful substances they contain, the authors aim to raise awareness and support better food policies. These findings can inform future research and public health efforts aimed at reducing cancer risk through healthier eating habits.

## 1. Introduction

Cancer remains a major contributor to global morbidity and mortality, with its burden steadily increasing in recent decades. According to the GLOBOCAN 2022 report, there were approximately 20 million new cancer cases and 9.7 million deaths globally in 2022, with projections indicating a rise to 35 million cases by 2050 [1]. This growing burden reflects the complex nature of cancer, which arises from an intricate interplay of genetic predispositions and a wide range of environmental factors. Among these, dietary exposures—particularly the consumption of certain types of preserved and processed foods—have been consistently associated with increased risks of specific cancers, including colorectal and esophageal cancer [2,3]. These factors span demographic, socioeconomic, lifestyle, and healthcare-related dimensions, with significant disparities observed across regions and income levels [4,5].

Notably, low- and middle-income countries (LMICs) are experiencing the fastest rise in cancer incidence, now accounting for over two-thirds of global cancer deaths [5,6]. This disproportionate impact is closely linked to the increasing adoption of Westernized lifestyles in these regions, characterized by tobacco use, sedentary behavior, and diets high in processed, calorie-dense foods—all of which are known contributors to cancer risk [4]. While sedentary behavior has been independently associated with increased cancer incidence, particularly for colon, endometrial, and lung cancers [7], there is growing evidence that ultra-processed foods contribute to cancer risk through distinct mechanisms such as chronic inflammation, metabolic dysregulation, and exposure to potentially carcinogenic additives and neo-formed contaminants [8,9]. These factors may act synergistically but also exert independent effects on cancer development, underscoring the importance of examining their contributions separately.

While the carcinogenic effects of tobacco use [10,11,12,13] and alcohol consumption [11,12] are well established in the literature, and the volume of related scientific publications has leveled off over time, dietary factors have garnered increasing attention from the research community. The relationship between diet and cancer is inherently complex, shaped by many interacting variables including nutritional composition, dietary patterns, cultural practices, and individual metabolic responses. This multifaceted nature has sustained and even intensified scholarly interest, leading to a notable increase in studies investigating dietary factors contributing to cancer in recent years [14].

Research on diet and cancer encompasses a broad spectrum of investigations, ranging from studies examining the carcinogenic effects of specific food components, additives, contaminants, and compounds formed during food processing [15,16,17,18] to analyses of the protective effects associated with complex dietary patterns. Notably, considerable attention has been given to the Mediterranean diet, which is widely recognized for its potential cancer-preventive properties due to its rich composition of fruits, vegetables, whole grains, healthy fats, and bioactive compounds [19,20,21,22].

More recently, however, researchers worldwide have increasingly focused on the harmful health effects of ultra-processed foods (UPFs), including their potential carcinogenic properties [23]. A growing body of evidence now links the higher consumption of UPFs to a broad spectrum of adverse health outcomes, with robust associations observed for increased risks of cancer, cardiometabolic diseases, and multimorbidity involving these conditions [24,25,26]. Notably, extensive multinational cohort studies have demonstrated that individuals with greater UPF intake face a significantly higher likelihood of developing cancer compared to those with lower consumption [27,28,29].

Studying UPFs and the risk of cancer requires an assumption that UPFs are defined within the NOVA classification [30] and they share specific characteristics [31] that are potentially carcinogenic.

Guided by the characteristics of some UPFs, this review provides a comprehensive overview of the evidence linking UPFs to cancer development, integrating mechanistic and epidemiological findings. By synthesizing current evidence and recent advancements in this field, we aim to identify critical areas for future research and inform public health strategies to mitigate the risks associated with UPF consumption.

## 2. Defining Ultra-Processed Foods: NOVA Classification and Core Characteristics

The term “ultra-processed food” was coined in 2009 [32], when researchers at the University of São Paulo, Brazil, developed a new food classification system [33]—later named NOVA [34]—as a response to the limitations of traditional food classification systems, such as the food pyramid [35]. While the food pyramid emphasized nutrients and food groups, it failed to address the health implications of modern food processing. NOVA introduced a paradigm shift by classifying foods based on the extent and purpose of industrial processing, allowing for a more accurate assessment of the relationship between food consumption patterns and chronic diseases, including cancer [30].

**Figure 1:**
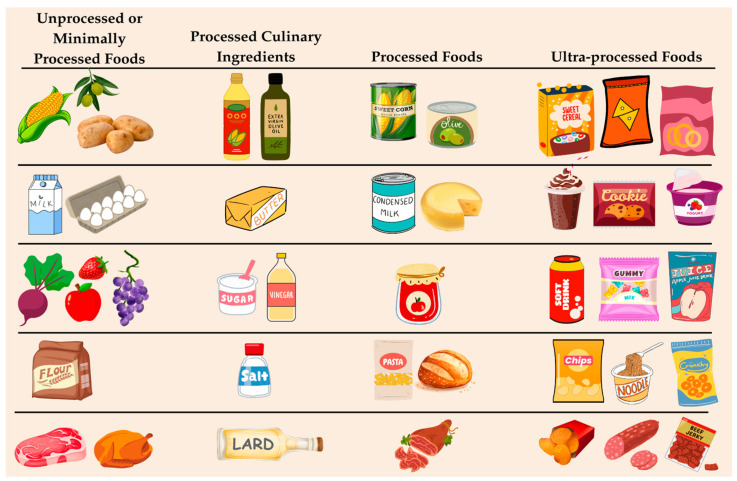
Classification of food groups according to NOVA.

NOVA divides foods into four groups: Group 1—Unprocessed or Minimally Processed Foods: These are edible parts of plants and animals (e.g., fruits, vegetables, grains, eggs, fresh meat) that may be cleaned, dried, frozen, or fermented without the addition of substances. Group 2—Processed Culinary Ingredients: Substances extracted from Group 1 foods or nature, including sugar, salt, oils, and fats, used in domestic cooking. Group 3—Processed Foods: Products made by combining Group 1 and Group 2 items, such as canned vegetables with salt, cheeses, and artisanal bread. These retain some recognizable characteristics of the original food. Group 4—Ultra-Processed Foods: Formulations made mostly or entirely from substances derived from foods, often containing little or no intact whole foods. Examples include soft drinks, packaged snacks, sweetened breakfast cereals, instant noodles, and reconstituted meat products [30] (see Figure 1).

UPFs are characterized by their convenience, hyperpalatability, affordability, extensive marketing, and widespread availability—qualities that make them staples of modern diets but also raise concerns about their health implications [25]. However, for this review, we focus on characteristics related to the composition, processing, and packaging of UPFs. Despite this analytical division, we emphasize that these features act synergistically and may contribute to cancer development through multiple biological pathways.

One of the most prominent features of UPFs is their adverse nutritional profile. These products are characterized by high levels of free sugars, sodium, saturated and trans fats, and energy density, while low in essential nutrients such as dietary fiber, high-quality protein, and micronutrients [36,37,38]. In addition to their poor nutritional profile [39], UPFs are primarily composed of ingredients not commonly used in home cooking. Rather than whole foods, they are formulated from refined and processed substances such as starches, protein isolates, hydrogenated oils, and high-fructose corn syrup [31,40]. These ingredients are combined with various cosmetic additives—substances added not for nutritional purposes but to enhance flavor, color, appearance, texture, or shelf life. Examples include artificial sweeteners (e.g., aspartame, acesulfame-K), emulsifiers (e.g., polysorbate-80, carboxymethylcellulose), preservatives (e.g., sodium nitrite), colorants (e.g., tartrazine), whitening agents (e.g., titanium dioxide), and flavor enhancers [41]. Additionally, UPFs may contain contaminants originating from packaging materials (e.g., bisphenols) [42], industrial processing (e.g., acrylamide) [43] or even from ingredients that are already contaminated, such as those containing human carcinogens (e.g., perfluoroalkyl substances) and potential carcinogens (e.g., glyphosate) [44,45] (see also Section 4. “Carcinogenic Compounds in UPFs”).

Despite some existing evidence, a controversy persists regarding the carcinogenic potential of certain ingredients and food additives used in the production of UPFs, as well as contaminants that may be formed or introduced during processing. These controversies stem from a range of factors, including ethical constraints in conducting human research, lobbying [46,47,48,49], and conflicts of interest [48] involving industries that produce these compounds. Moreover, carcinogenic risk assessments are typically conducted on individual compounds in isolation, which does not capture the real-life effects arising from the inevitable interactions, transformations, and synergies that occur among multiple compounds during the production of UPFs.

Beyond composition, the manufacturing process of UPFs raises additional concerns related to cancer. Industrial processing often involves physical and chemical techniques that significantly alter the food matrix [40]. High-temperature treatments, such as frying, baking, or extrusion, can lead to the formation of newly formed contaminants, including acrylamide, a well-known substance classified as a human carcinogen [43]. In contrast, foods in NOVA Groups 1 to 3 retain much of their original food structure, undergo milder processing, and do not generate or incorporate such levels of industrial contaminants.

In addition to compositional concerns and processing-related contaminants, the packaging of UPFs represents another critical source of potential exposure to carcinogenic compounds. These products are commonly stored in complex materials such as multilayer plastics, aluminum-based containers, or laminated pouches, designed to ensure a long shelf life and resistance to microbial contamination. However, such packaging can interact with the food, particularly when subjected to high temperatures during processing or over long storage periods. This interaction may result in the migration of endocrine-disrupting chemicals (EDCs) into the food. Conversely, foods in NOVA Groups 1 to 3 are typically packaged in simpler materials, such as glass, paper, or basic plastic, which are less likely to be exposed to conditions that promote chemical leaching.

**Figure 2:**
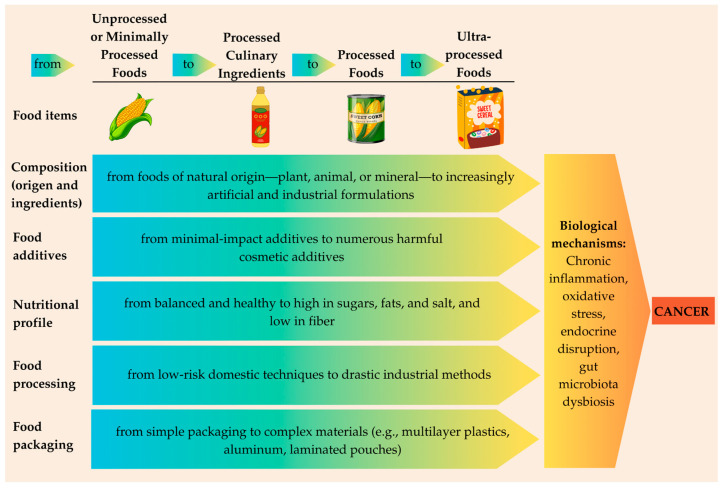
From unprocessed or minimally processed to ultra-processed foods: food processing intensity and its synergistic links to carcinogenesis.

Taken together, the unhealthy composition of UPFs, harmful processing methods, and exposure to packaging-derived contaminants operate synergistically to create an environment conducive to carcinogenesis. These factors likely interact through overlapping mechanisms, such as chronic inflammation, oxidative stress, endocrine disruption, and alterations in gut microbiota. Across the NOVA classification, there is a progressive intensification from Group 1 to Group 4 in terms of processing, additive content, and packaging complexity. This transition reflects a shift from foods of natural origin—plants, animals, or minerals—to increasingly artificial and industrial formulations. In contrast, foods in the other NOVA groups, such as unprocessed or minimally processed foods, maintain their natural characteristics and have been part of human diets without significant health concerns for centuries (see Figure 2). Drawing an analogy with cancer biology, while UPFs might be likened to mutated cells with unpredictable impacts on bodily functions, traditional foods resemble normal cells that reliably fulfill their roles.

## 3. Molecular Mechanisms Linking UPFs to Inflammatory Diseases

The consumption of UPFs has been strongly associated with metabolic disturbances, including obesity, systemic inflammation, and insulin resistance [50,51,52,53], and an ultimately increased risk of several cancers, including colorectal [2,54], breast [25], liver [28] as well as other hormone-related cancers [55] (see also Section 5. “Epidemiological Evidence Linking UPFs to Cancer”).

The nutritional profile and structural characteristics of UPFs have been linked to cellular alterations that contribute to oxidative stress, which in turn affects immune cell proliferation, apoptosis, and signaling pathways [56]. The consumption of UPFs promotes low-grade, chronic inflammation across age groups, a condition recognized as a key risk factor for several non-communicable diseases, including cancer and cardiometabolic disorders [57,58,59]. Inflammatory responses can disrupt gut microbiota composition and function, further increasing disease susceptibility [58,60]. These inflammatory signals may contribute to carcinogenesis by enhancing oxidative stress, activating pro-survival and anti-apoptotic pathways in epithelial cells, and creating a microenvironment conducive to tumor growth, angiogenesis, migration, and invasion [56]. Moreover, the degraded physical structure of UPFs can affect absorption kinetics, satiety, glycemic response, and microbiota dynamics, compounding their impact on chronic disease risk. Accordingly, preclinical and clinical research have increasingly focused on understanding how food processing and formulation contribute to the etiology of chronic inflammatory diseases [61].

The environment created in the gut by UPFs, a hallmark of the Western diet, is an evolutionarily unique selection ground for microbes that can promote diverse forms of inflammatory disease [62,63]. The relatively rapid shift from consuming pre-agricultural wild foods for thousands of years to consuming postindustrial semi-processed and ultra-processed foods endemic to the Western world less than 200 years ago did not allow for adequate adaptation in human physiology. There is increasing evidence of an association between diets rich in UPFs and gut disease, including inflammatory bowel disease, colorectal cancer, and irritable bowel syndrome, with food additives in UPFs shown to affect gut health [64]. A recent study assessed the cross-sectional associations between UPF consumption and gut microbiota in 10 senior subjects. It determined the taxonomic analysis of the fecal microbiota, uncovering a significant positive association between specific taxa (*Alloprevotella*, *Negativibacillus*, *Prevotella*), UPF consumption, inflammatory gastro-intestinal diseases, and a low consumption of fruits and vegetables [65]. Moreover, UPFs may influence microbiota composition in children during their first year of life [66] and have a differential impact on women and men [67]. Clinical trials on UPFs and gut microbiota composition are ongoing [68]. The environment created in the gut by UPFs is an evolutionarily unique selection ground for microbes that can promote diverse forms of inflammatory disease, increasing the risk of gastrointestinal health concerns like inflammatory bowel disease, neurodegenerative diseases, and metabolic health consequences, including obesity.

UPFs have an impact on several key biological pathways, including altered serum lipid concentrations, oxidative stress, dysglycemia, insulin resistance, and hypertension. Compared to minimally processed foods, UPFs tend to have a higher glycemic impact on average [69], which may exacerbate metabolic disturbances. Furthermore, many commercially manufactured UPFs contain industrial trans fatty acids—found in partially hydrogenated vegetable oils—which negatively affect blood lipoprotein profiles and increase the risk of cardiovascular diseases [70]. Prospective cohort studies in numerous countries have shown that UPF consumption leads to the development of incident hypertriglyceridemia, low HDL cholesterol, and high LDL cholesterol [67,68,69,70], as well as diabetes [71,72,73,74] and hypertension [71,72,73,74,75].

Food additives and newly formed contaminants produced during processing may also contribute to the risk of cardiovascular disease [76]. Research suggests that ultra-processed foods may affect cardiometabolic health through myriads of mechanisms, beyond the traditionally recognized individual nutrients. Processing induces significant changes to the food matrix, for which UPFs may affect health outcomes differently than unrefined whole foods with similar nutritional composition [76].

Another reason that UPFs are concerning is that they often contain carcinogenic components that result from food packaging. As we have already mentioned, the packaging materials of UPFs may contain endocrine-disrupting chemicals, such as bisphenol A. This substance is structurally similar to 17β-estradiol, and it has been shown to promote insulin resistance, oxidative stress, inflammation, adipogenesis, and pancreatic B-cell dysfunction by binding to estrogen-related receptors [77]. Additionally, phthalates, another group of chemicals used in food packaging, can also migrate into food products and have been associated with adverse health effects, including endocrine disruption and carcinogenesis [42]. The presence of these harmful substances in packaging materials highlights the need for stricter regulations and safer alternatives to minimize the risk of cancer associated with UPFs.

Given these multifactorial mechanisms, understanding how UPF consumption translates into measurable cancer risk at the population level is essential.

## 4. Carcinogenic Compounds in UPFs

Mutagens are chemical compounds capable of damaging DNA [78]. Given that UPFs are produced industrially, primarily using chemically derived ingredients, there is growing concern about the potential health effects of both the ingredients used and the substances formed during their processing and packaging, including carcinogenic effects. In this section, we expand on some of the compounds mentioned earlier and introduce additional ones relevant to this discussion.

Regarding the composition of UPFs, food additives are among the components of most significant concerns for several reasons: regulations governing their use vary across countries; the quantities of additives present in foods are not disclosed on nutrition labels; and there is limited oversight of additive use by the food industry. Moreover, the synergistic effects between additives and other ingredients in the development of cancers and other diseases remain poorly understood [79]. Despite significant efforts to assure safety, the toxicological analysis of these substances generally relies on their direct toxicity to target organs (liver and kidney) or their genotoxic effects. Much less attention is paid to the effects of these compounds on cells of the immune system [80]. Many studies have demonstrated that the use of synthetic preservatives and chemical additives in food is causing poisoning, cancer, and other degenerative disorders. For example, an early study has shown that exposure to the food color tartrazine, the preservatives sodium nitrate and sodium benzoate, and the antioxidant BHT significantly increased DNA content in the protozoan Tetrahymena pyriformis, which was used as a toxicological model [81]. This was concerning, since mitogenic stimuli substantially alter susceptibility to chemical carcinogenesis [81]. New solutions for food preservation with quality maintenance are needed and are currently emerging [82].

Mutagens can also enter the body through the consumption of improperly cooked or processed food products, particularly those that have been subjected to high temperatures or prolonged cooking times. For example, the thermal processing and smoking of meat can generate carcinogenic substances, such as heterocyclic amines (HCAs), polycyclic aromatic hydrocarbons (PAHs), N-nitroso compounds (NOCs), and monochloropropane diols and their esters, which are toxic and carcinogenic [83,84]. These carcinogenic components contribute to the overall risk associated with consuming UPFs [85]. The formation of these carcinogenic compounds is influenced by factors such as heat, moisture, and the sugar/lipid content of the food [86]. Additionally, the Maillard reaction, sugar reduction, thermal degradation of polyphenols, and lipid oxidation during high-temperature processing can produce neo-formed contaminants (NFCs) like acrylamide, furan, furfuryl alcohol, and hydroxymethylfurfural, that when absorbed by the body, can be converted into metabolites that cause genotoxicity, carcinogenicity, and hepatoxicity [43].

Another concern regarding processed meats is the presence of sodium nitrate, a commonly used preservative that can lead to the formation of nitrosamines in the stomach. Diethylnitrosamine, a type of nitrosamine, is a mutagenic and genotoxic agent that causes DNA alterations and gene expression changes, leading to liver cancer in experimental wild-type or transgenic animal models [87]. Furthermore, food products with high fat and protein content are more prone to mutagenic formation [88].

The consumption of processed meat has been classified as directly carcinogenic for humans by the International Agency for Research on Cancer [89]. Despite efforts to prevent the formation of these compounds during processing, eliminating them is challenging due to their complex formation mechanisms. Understanding these mechanisms is crucial for developing strategies to minimize their impact on human health [84].

Additionally, UPFs may contain contaminants originating from packaging materials. For example, BPA, a synthetic organic compound used in epoxy linings of cans, and phthalates, used as plasticizers, can leach into food and beverages, especially when exposed to heat, and have been associated with a range of health issues, including cancer [90]. Additionally, phthalates, another group of chemicals used in food packaging, can also migrate into food products and have been associated with adverse health effects, including endocrine disruption and carcinogenesis [42]. Moreover, organophosphate esters (OPEs)—commonly used as flame retardants and plasticizers, particularly in self-heating meal packaging—have been linked to alterations in thyroid function and an increased risk of thyroid cancer [91,92].

Various factors, including the presence of harmful additives and preservatives, the formation of carcinogens during processing, and the leaching of toxic substances from packaging materials, influence the carcinogenic potential of UPFs.

The carcinogenic potential of compounds found in UPFs depends not only on their intrinsic toxicity but also on their concentration and frequency of exposure. Some substances, such as acrylamide, have demonstrated carcinogenicity in animal models at relatively high doses; however, the levels typically found in food are considered to be low, and their impact on human cancer risk remains uncertain and likely modest at habitual dietary exposures [93]. Conversely, nitrosamines, particularly N-nitrosodimethylamine (NDMA), have shown strong carcinogenic effects even at low concentrations and are present in certain processed meats at levels that raise concern, leading regulatory agencies to recommend minimizing exposure as much as possible [94]. Therefore, while some compounds may be present in trace amounts unlikely to pose a significant health threat, others can exert biologically relevant effects even at low doses, underscoring the need for strict monitoring and risk assessment based on both toxicological data and real-world consumption patterns.

This variability highlights the importance of considering not only the presence of carcinogens in UPFs, but also how their biological effects scale with exposure. The risk of carcinogenesis associated with these compounds is strongly influenced by their dose–response relationships, which can follow linear or nonlinear patterns depending on the substance, its chemical structure, mode of exposure, and underlying biological mechanisms. Understanding these dynamics is essential for accurately assessing cancer risk and establishing practical safety guidelines. Further research is needed to explore safer alternatives and improve regulations to protect consumers from the potential carcinogenic effects of UPFs.

## 5. Epidemiological Evidence Linking UPFs to Cancer

As stated above, the consumption of UPFs has been increasingly scrutinized for its potential link to cancer risk. Population-based studies have provided a foundation for understanding this association. For instance, a 2023 comprehensive systematic review and meta-analysis identified a total of 13 studies (4 cohort studies and 9 case-control studies), with a total of 625,738 participants, utilizing fixed-effects or random-effects models to pool data [55]. This study suggests a correlation between high UPF consumption and increased cancer risk, especially in the digestive tract and some hormone-related cancers. However, the results remain inconclusive for other types of cancer. Another systematic review and dose–response meta-analysis aimed to clarify the relationship between UPF consumption and breast cancer risk, including six articles that involved 462,292 participants. It found that a higher consumption of UPFs is slightly related to a higher risk of breast cancer, warranting that extensive prospective cohort studies are warranted to confirm these results [95].

The NOVA classification system has been instrumental in evaluating the association between UPF consumption and cancer risk. A systematic literature review using this system identified observational studies that investigated this relationship: eleven reports were identified, including eight retrospective case-control studies and three prospective cohorts [96]. The outcome was the risk of total cancer and/or one or more of colorectal, breast, prostate, pancreatic, chronic lymphocytic leukemia, and central nervous system tumors. The available suggestive evidence showed a consistent significant association between the intake of UPFs and the risk of overall and several cancers, including colorectal, breast, and pancreatic cancer [96].

Prospective cohort studies have also contributed to the body of evidence regarding the connection between UPF consumption and cancer risk. Five prospective cohort studies, comprising 1,128,243 participants (241,201 participants in the highest and 223,366 in the lowest levels of UPF consumption), compared the highest and lowest levels of UPF consumption according to the NOVA food classification and reported the risk of gastrointestinal cancers by subsite. The mean follow-up ranged from 5.4 to 28 years. The highest UPF consumption was significantly associated with an increased risk of colorectal cancer, colon cancer, and non-cardia gastric cancer compared with the lowest UPF intake. However, no association was found between high UPF consumption and hepatocellular, esophageal, pancreatic, gastric cardia, and rectal cancer [97].

An ongoing debate focuses on whether the detrimental effects are due to the ultra-processing itself or the lower nutritional quality of UPFs. Meta-analyses and systematic reviews have consolidated evidence on single constituents of UPFs, such as trans-fatty acids. Despite heterogeneity, a higher risk of prostate and colorectal cancer by a high consumption of trans-fatty acids was found [98]. Maintaining an optimal acid-base balance is crucial for maintaining good health. Dietary acid load (DAL) is a measure of the acid load derived from diet, taking into account both the potential renal acid load from food components like protein, potassium, phosphorus, calcium, and magnesium, and the organic acids from foods, which are metabolized to bicarbonate and thus have an alkalinizing effect. Current UPFs are characterized by a high DAL, due to large amounts of animal protein and processed foods. A large-scale prospective cohort study further identified an increased risk of overall cancer incidence in individuals adhering to a high DAL/UPF diet, with every 10% increment of adherence increasing the overall cancer incidence by 2% and overall mortality by 6% [99].

Systematic reviews and meta-analyses have also explored the broader impact of UPFs consumption on noncommunicable diseases, with a broader conception, including cancer. Recently, a study investigated the association between the consumption of UPFs and the risk of non-communicable diseases, morbidity, and mortality. Forty-three observational studies were included (N = 891,723): 21 cross-sectional, 19 prospective, 2 case-controls, and 1 that conducted both a prospective and cross-sectional analysis. Meta-analysis demonstrated that the consumption of UPFs was associated with an increased risk of obesity, all-cause mortality, metabolic syndrome, and depression; in addition, consumption of UPFs was associated with cardiometabolic diseases, frailty, and irritable bowel syndrome [53].

Altogether, these studies underscore the potential public health implications of UPF consumption. The epidemiological evidence linking UPF consumption to cancer risk is substantial but not yet definitive. Population-based studies, case-control, and cohort studies, and meta-analyses have all contributed to this growing body of evidence. While there is a clear indication of an association between high UPF consumption and increased cancer risk, further high-quality epidemiologic and mechanistic investigations are needed to establish causality and understand the underlying mechanisms. The current evidence highlights the importance of dietary guidelines that limit UPF consumption to reduce cancer risk and enhance overall public health.

## 6. Future Directions

Given the growing body of evidence linking UPFs to cancer and other health issues, preventive strategies to reduce their consumption are urgently needed. Dietary recommendations from health organizations consistently emphasize the importance of limiting UPF intake as a fundamental preventive measure [100].

These recommendations should be implemented across all sectors, including both public and private sectors, as well as healthcare and educational institutions. This task should not be limited to health professionals but should also involve other qualified professionals committed to promoting public health.

Regulatory policies and clear food labeling play a central role in controlling the consumption of UPFs. Several countries have successfully introduced regulations on the marketing and sale of these products. However, significant efforts are still needed to overcome the barriers that countries face in regulating food marketing [101].

Building on current regulatory efforts, urgent advances are needed to regulate not only the use of food additives but also the maximum permitted quantities per serving, as well as the transparent display of these amounts on nutrition labels.

Public education and awareness programs are key components of a comprehensive strategy to mitigate the cancer risk associated with UPFs. Informing the population about the health risks linked to UPF consumption through improved food labeling, public health campaigns, and school-based nutrition education can significantly promote healthier eating habits. This effort should be part of a broader, multifaceted approach that combines dietary recommendations, regulatory frameworks, product reformulation, and investments in health literacy to support the transitions toward minimally processed, healthier diets.

## 7. Conclusions

The growing body of evidence linking UPFs to cancer risk reflects an urgent need to reassess global dietary patterns and the food systems that support them. This review has synthesized epidemiological findings, mechanistic hypotheses, and public health implications related to UPF consumption and cancer. The analysis reveals consistent associations between a high UPF intake and an increased risk of various cancer types, particularly colorectal, breast, and overall cancer incidence and mortality. Potential biological mechanisms, ranging from poor nutritional quality and the presence of harmful additives to processing-induced contaminants and the disruption of the gut microbiota, offer plausible explanations for these associations.

To mitigate the health risks posed by UPFs, a multifaceted approach is required. This includes establishing robust regulatory frameworks, refining food labeling practices, and implementing evidence-based dietary guidelines that prioritize the consumption of minimally processed foods. Education and awareness campaigns should play a central role in informing the public about the risks of UPFs and promoting healthier food choices. At the healthcare and community level, cancer prevention programs should incorporate dietary counseling that emphasizes minimally processed, plant-based foods, while educational campaigns must raise awareness about the risks of UPFs beyond nutritional content alone. Furthermore, dismantling food industry lobbies and eliminating conflicts of interest in policy making are essential steps toward prioritizing public health over corporate profit.

It is essential to emphasize that the health risks associated with ultra-processed foods (UPFs) cannot be solely attributed to their poor nutritional quality. While their excessive contents in terms of added sugars, sodium, and unhealthy fats is undoubtedly harmful, the potential for adverse health effects extends far beyond these aspects. The synergy between these components and various substances, either added intentionally during processing, may enhance their detrimental impact. The concept of ultra-processed foods encompasses not only the level of processing but also the industrial complexity of their formulations, which are specifically engineered to be hyper-palatable and difficult to resist. This ultracomplexity, driven by multiple stages of physical, chemical, and biological manipulation, plays a central role in shaping the unique and concerning profile of UPFs in terms of health outcomes.

Finally, the findings underscore a paradigm shift in nutritional epidemiology, as concerns over UPFs catalyze a broader reevaluation of modern diets and industrial food production. While preventive actions are crucial, ongoing research is needed to elucidate the carcinogenic mechanisms of specific substances in UPFs and their potential synergistic effects within the human body. Only through coordinated efforts in research, regulation, education, and advocacy can we effectively reduce UPF consumption and its growing impact on the global cancer burden.

## Abbreviations

The following abbreviations are used in this manuscript:

DAL	Dietary acid load
EDCs	Endocrine-disrupting chemicals
HCAs	Heterocyclic amines
LMICs	Low- and middle-income countries
NDMA	N-nitrosodimethylamine
PAHs	Polycyclic aromatic hydrocarbons
UPFs	Ultra-processed foods
